# Results of the implementation of target zero program for surgical site infections in a middle-sized private hospital in Southern Brazil

**DOI:** 10.1186/2047-2994-4-S1-P83

**Published:** 2015-06-16

**Authors:** CC Ponzi, C Pompermayer

**Affiliations:** 1 Hospital Unimed Chapecó, Chapecó, Brazil

## Introduction

Surgical site infections (SSI) are preventable events that increase hospital length of stay, risk of death and costs. Target Zero Program (TZP) was implemented in order to diminish the rates of SSI in surgical procedures to below 1% in a medium-sized private hospital in Southern Brazil.

## Objectives

To prospectively analyse the impact of a TZP on reducing SSI.

## Methods

SSI rates were measured from Jan. 2012 to Dec. 2014, and the TZP was implemented in May 2014. The taken interventions were as follows: pre-admission (adequate nutrition, stopping smoking, treating pre-existing infections and compensating pre-existing comorbidities), pre-surgical procedure (2% chlorhexidine bathing, trichotomy if necessary, adequate hand washing by staff, adequate patient´s skin degermation and appropriate antibiotic administration 30-60 minutes prior to incision), trans-surgical procedure (*core temperature between 36-37*,*7°C*, *adequate hemostasis*, *maintenance of normal blood pressure and limiting circulation of persons into the operating room*) and post-surgery (*adequate wound dressing*, *adequate management of drains and hand hygiene*)*.*

## Results

8.975 surgical procedures were analyzed from Jan 2012 to Dec 2014, and the mean SSI rate was 1.31%, for all surgical wounds (clean, potentially contaminated and contaminated). Baseline data showed a SSI rate of 1.6% (1.4% for clean wounds and 1.15% for potentially contaminated wounds) TZP was started in May 2014 and reassessed in July 2014. Overall SSI rates dropped from 1.62% in 2012 and 1.58% in 2013 to 0.89% in 2014. SSI in clean wounds were 2.65% in 2012, 1.83% in 2013 and 0.99% in 2014, and SSI rates in potentially contaminated wounds were 0.98% in 2012, 1.35% in 2013 and 0.78% in 2014.

When the Target Zero Program was reassessed in July 2014 SSI rates declined from 1.92% to 0.59% (mean 0.61%) overall by December 2014, from 2.84% to 0.55% (mean 0.75%) for clean wound surgeries (an 80.63% drop) and there was no significant difference for potentially contaminated wounds.

## Conclusion

SSI are preventable events and can be reduced with low-cost bundles of interventions, and reassuring staff and surgeons about compliance and engagement with multiple interventions may result in even more significant declines, resulting in substantial cost savings.

## Disclosure of interest

None declared.

